# Analysis of [*Gossypium capitis-viridis* × (*G*.*hirsutum* × *G*.*australe*)^2^] Trispecific Hybrid and Selected Characteristics

**DOI:** 10.1371/journal.pone.0127023

**Published:** 2015-06-02

**Authors:** Di Chen, Yuxiang Wu, Xiling Zhang, Fuguang Li

**Affiliations:** 1 Cotton Research Institute, Chinese Academy of Agricultural Science, Anyang, Henan, China; 2 Department of Agriculture, Shanxi Agricultural University, Taigu, Shanxi, China; Zhejiang University, CHINA

## Abstract

Speciation is always a contentious and challenging issue following with the presence of gene flow. In *Gossypium*, there are many valuable resources and wild diploid cotton especially C and B genome species possess some excellent traits which cultivated cotton always lacks. In order to explore character transferring rule from wild cotton to upland tetraploid cotton, the [*G*. *capitis-viridis* × (*G*. *hirsutum* × *G*. *australe*)^2^] triple hybrid was synthesized by interspecies hybridization and chromosome doubling. Morphology comparisons were measured among this hybrid and its parents. It showed that trispecific hybrid F_1_ had some intermediate morphological characters like leaf style between its parents and some different characters from its parents, like crawl growth characteristics and two kind flower color. It is highly resistant to insects comparing with other cotton species by four year field investigation. By cytogenetic analysis, triple hybrid was further confirmed by meiosis behavior of pollen mother cells. Comparing with regular meiosis of its three parents, it was distinguished by the occurrence of polyads with various numbers of unbalanced microspores and finally generating various abnormal pollen grains. All this phenomenon results in the sterility of this hybrid. This hybrid was further identified by SSR marker from DNA molecular level. It showed that 98 selected polymorphism primers amplified effective bands in this hybrids and its parents. The genetic proportion of three parents in this hybrid is 47.8% from *G*. *hirsutum*, 14.3% from *G*. *australe*, 7.0% from *G*. *capitis-viridis*, and 30.9% recombination bands respectively. It was testified that wild genetic material has been transferred into cultivated cotton and this new germplasm can be incorporated into cotton breeding program.

## Introduction

There are abundant germplasms in *Gossypium* including approximately 46 diploid and 6 polyploid species (AADD, 2n = 4x = 52) [1–3]. Diploid wild cotton especially C and B genome species has some excellent traits which are devoid of in cultivated cotton, especially *G*. *capitis-viridis* with high quality fiber and a strong resistance to *Verticillium* and *Fusarium* wilt, *G*. *australe* with the glandless-seed and glanded-plant trait. These Australian cottons of C and B genomes are however phylogenetically remote from upland cotton. The only glanded plants issued from glandless seeds produced this way were hexaploid, pentaploid and multiple addition materials [4] by transformation from Australian cottons to upland cotton. A cotton mutant totally devoid of pigment glands discovered by McMichael [5] and subsequently used in several breeding programs to create cotton commercial varieties with gossypol-free cotton seed. Biotechnological methods such as embryo rescue/ovule culture or interspecific cell fusion are also used to overcome sexual barriers between the cultivated and wild cottons [6]. Yu et al [7] generated fertile somatic hybrids between tetraploid upland cotton and wild cotton *G*. *trilobum* by symmetric electrofusion and this hybrid showed strong photosynthesis. The [(*G*. *hirsutum*×*G*. *raimondii*)^2^ ×*G*. *sturtianum*] triple hybrid was synthesized for selecting genotypes of *G*. *hirsutum* genetically balanced and expressing the low gossypol seed and high-gossypol plant trait [4].

*G*. *hirsutum* is the most important cultivated cotton for its fiber in the world. Wang et al [8] recently created a draft sequence of the putative D-genome parent, *G*. *raimondii*. It is so beneficial for studying cultivated polyploid genomes and to further explore its genome evolution and subgenomes interaction. What is needed is an efficient means of incorporating this diverse and divergent genetic material into upland cotton breeding programs. The present study was initiated in order to monitor the introgression of chromosome segments from the wild species *G*. *australe*, *G*. *capitis-viridis* to cultivated cotton and explore how genomes adjust when they come into contact with each other.

## Materials and Methods

### Plant materials

We chose *G*. *hirsutum* TM-1, *G*. *australe*, *G*. *capitis-viridis* as materials. *G*. *hirsutum* is an cultivated tetraploid cotton with genome [AD]_1_ (2n = 4x = 52), while *G*. *australe* and *G*. *capitis-viridis* are wild diploid cotton species with the genome C and B (2n = 2x = 26). All the wild paternal pollen used for the creation of the trispecific hybrid CHA [*G*. *capitis-viridis* ×(*G*. *hirsutum* ×*G*. *australe*)^2^] in this work, were kindly supplied by the cotton collection of National Wild Cotton Nursery in Sanya of China.

### Triple hybrid synthensis

Tetraploid *G*. *hirsutum* TM-1 was first crossed directly with the diploid parents *G*. *australe*, creating a triploid hybrid [ADC]. Allohexaploid was further synthesized by colchicine-doubling the sterile intergenomic hybrid *G*. *hirsutum* × *G*. *australe*. Allopolyploid trispecific CHA hybrid [genome BADC] was finally obtained by allohexaploid crossing with the other wild diploid maternal parent *G*. *capitis-viridis* according to the pseudophyletic introgression method [9].

### Morphology characteristic observation

In every growth period of this hybrid, morphology characteristics were observed from its whole plant to leaf style, flower color and performances, pollen grain morphology, its seed set and fertility. Comparisons from different characteristic and performance for this hybrid were also made with its parents.

### Meiotic analyses

Immature flower buds in different development stages were collected from three parents and this triple hybrid, fixed between 8.00–11.00 am in freshly prepared Carnoy’s solution (ethanol: acetic acid = 3:1) for 24 h, and then stored at 4°C in 70% EtOH for future studies. Meiotic analysis were carried out on suitable size flower buds. After washing the fixed buds in distilled water, anther squashes were stained on slides in Carbol fuchsin solution and observed under a light microscope. Photographs were taken from freshly prepared slides using an Olympus BX60 microscope with automatic camera. Size and sterility of pollen grains from randomly selected anthers were also studied staining with Carbol fuchsin solution. Interpretations were based on analyses of between 15–50 pollen mother cells from the triple hybrid.

### SSR molecular identification

The hybrid identity and its parents was also confirmed by SSR molecular markers. SSR analysis was examined by *G*. *hirsutum*, *G*. *australe*, allohexaploid, this hybrid and *G*. *capitis-viridis*. DNA from each plant was extracted from young leaves using CTAB method and quantified using agarose gel electrophoresis. DNA amplification was carried out in a volume of 10 μL containing 1 μL 10×buffer, 1.6 μL Mgcl_2_ (25 mM), 0.5 μL dNTPs (10mM), 2 μL template DNA (50 ng μL^-1^), 0.8 μL primers (2.5 μM) (0.4 μL of forward and reverse primer each), 0.1 μL Taq polymerase (5 U/μL, Sangon), 5.6 μL ddH_2_O. PCR was programmed with an initial denaturing for 3 minutes at 95°C; followed by 30 cycles of pre-denaturation for 3 minute at 94°C; denaturation for 40 s at 94°C; annealing at 57°C for 45 s; extension for 1 minutes at 72°C, and a final extension for 5 minutes at 72°C in a DNA Mastercycler, Eppendorf, Germany. PCR product (10 μl) was resolved in PAGE by silver staining for the resolving of the bands.

## Results

### Morphology characteristic of this triple hybrid

**Fig 1:**
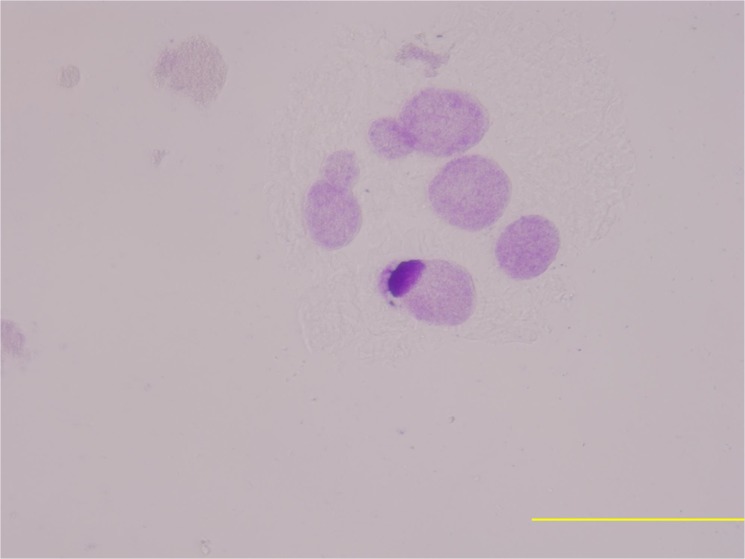
Whole semi-bush plant with crawl growth characteristic and long epidermis hair in the stem, heart and thick leaf style of this triple hybrid.

**Fig 2:**
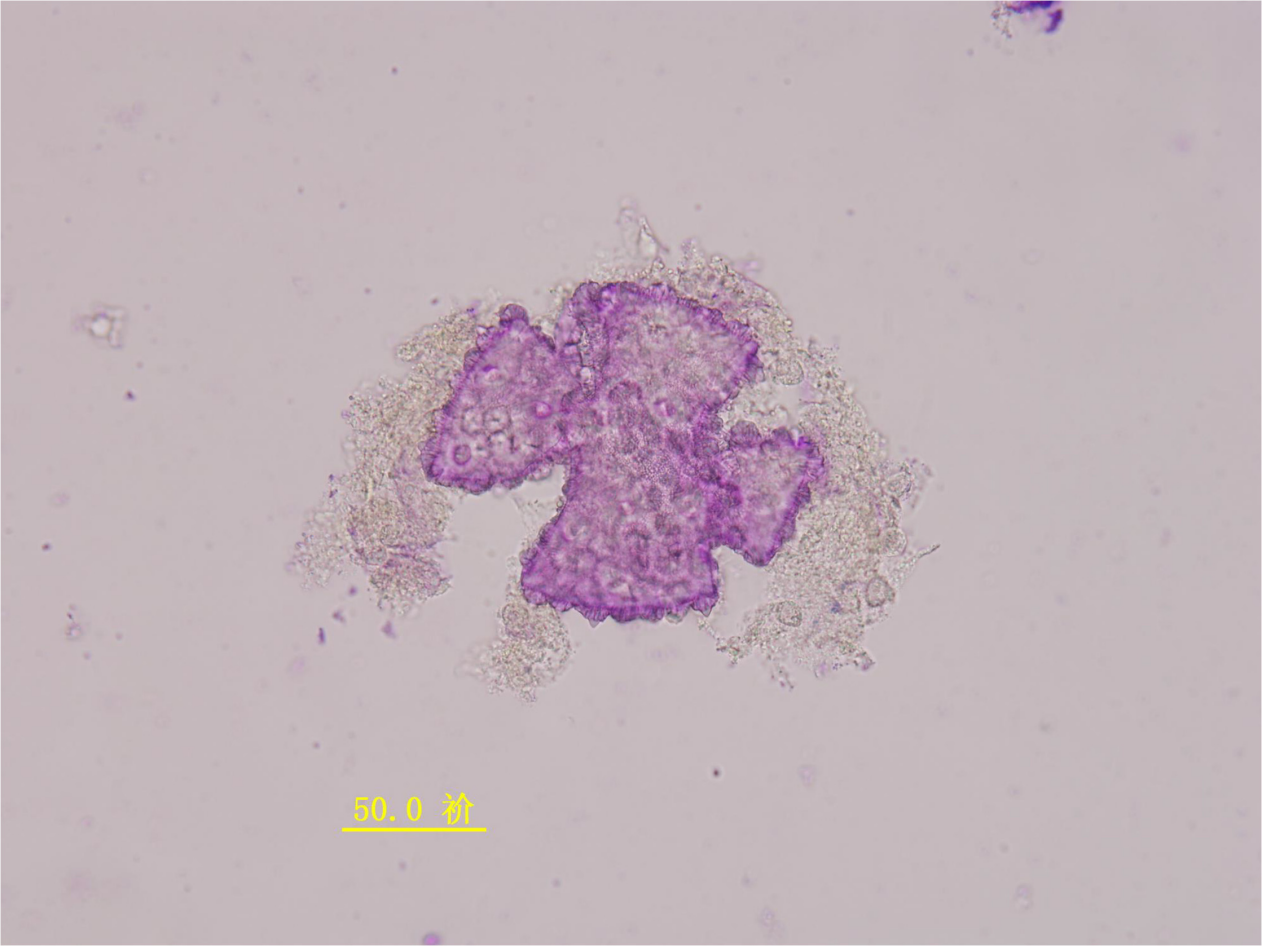
Flowers with two kind color of this triple hybrid at the same time (pink and white) and the stigma and stamen with yellow pollen grains of this hybrid.

**Fig 3:**
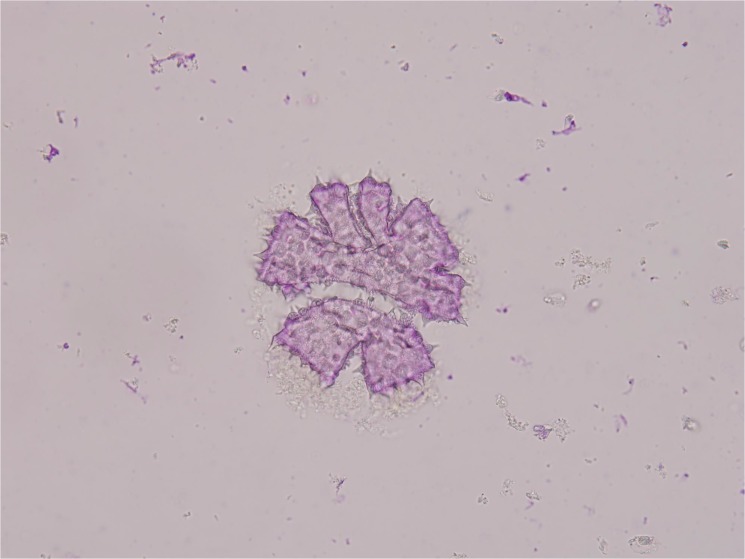
Flower characteristic and leaf performance comparison for this hybrid with three parents (from up to down: *G*. *hirsutum*, *G*. *australe*, allohexaploid, *G. capitis-viridis* and triple hybrid respectively).

The hybrid plants were perennial, semi-bush with crawl growth characteristic different from any other parents (Fig 1). It has long epidermis hair in whole plant especially on the stem and two side of leaf following with its wild-type parents *G*. *australe* (Fig 1). The shape of leaves is heart style with round edge which is intermediate character between its parents and thicker also different from its three parents (Fig 1). It produced many flower buds, and flowered by pink or white petal with unclear or no speck, sometimes pink and white flower at the same day (Fig 2). It had normal stigma and stamen with yellow pollen grains like its upland parents (Fig 2). But most pollen grains were abnormal resulting in its sterility. For several years this hybrid had no seed set. Flower performance and leaf characteristic for this hybrid were also evaluated and compared with its three parents in Table 1 and Fig 3.

**Table 1:** Accessions in this study and its characteristics.

Accessions	Leaf	Flower
*G*. *hirsutum*	Big and green	Big and yellow
*G*. *australe*	Small and light grey	Purple with dark speck
Allohexaploid	Heart style and grey	Big and red
BADC hybrid	Heart style and thicker	White or pink
*G*. *capitis-viridis*	Grey with deep divided	Light purple with dark speck

The triple hybrid had long epidermis hair on the leaf and stem inheriting from its wild parents. It is highly resistant to insects comparing with other cotton species which always have some damage from cotton worm. In Sanya of China, cotton can grow in whole year because of comfortable hot climate. So cotton is very easy to be harmed by various insects. By four year field investigation from 2010–2014, it was tested that some cultivated cotton species like TM-1, CCRI 12, CCRI 16 were all damaged mostly by various insects like pink worm, cotton bollworm, aphid, thrips, mite, leaf hopper and stinkbug, whereas this triple hybrid performed high insect resistance especially immune to pink worm, cotton bollworm, aphid and mite under no any protection control. The possible reason is that it inherits the resistant gene from its parents *G*. *capitis-viridis* and *G*. *australe*. We will further confirm its resistant characteristics and identify this resistant gene in next study. The triple hybrid is also high drought resisting because of its long epidermis hair on the leaf and stem.

In addition, the hybrid plants were perennial, semi-bush with crawl growth characteristic different from any other parents. So it can prevent from wind damage suitable for cultivating dwarf and wind resistant species in Hainan of China.

We try to backcross this hybrid with cultivars and hope to recover its fertility incorporating into next cotton breeding program. We will continue to examine the stability of the morphological features and further to check its other excellent agronomic traits of this hybrid in our later study.

### Meiotic Analyses

**Fig 4:**
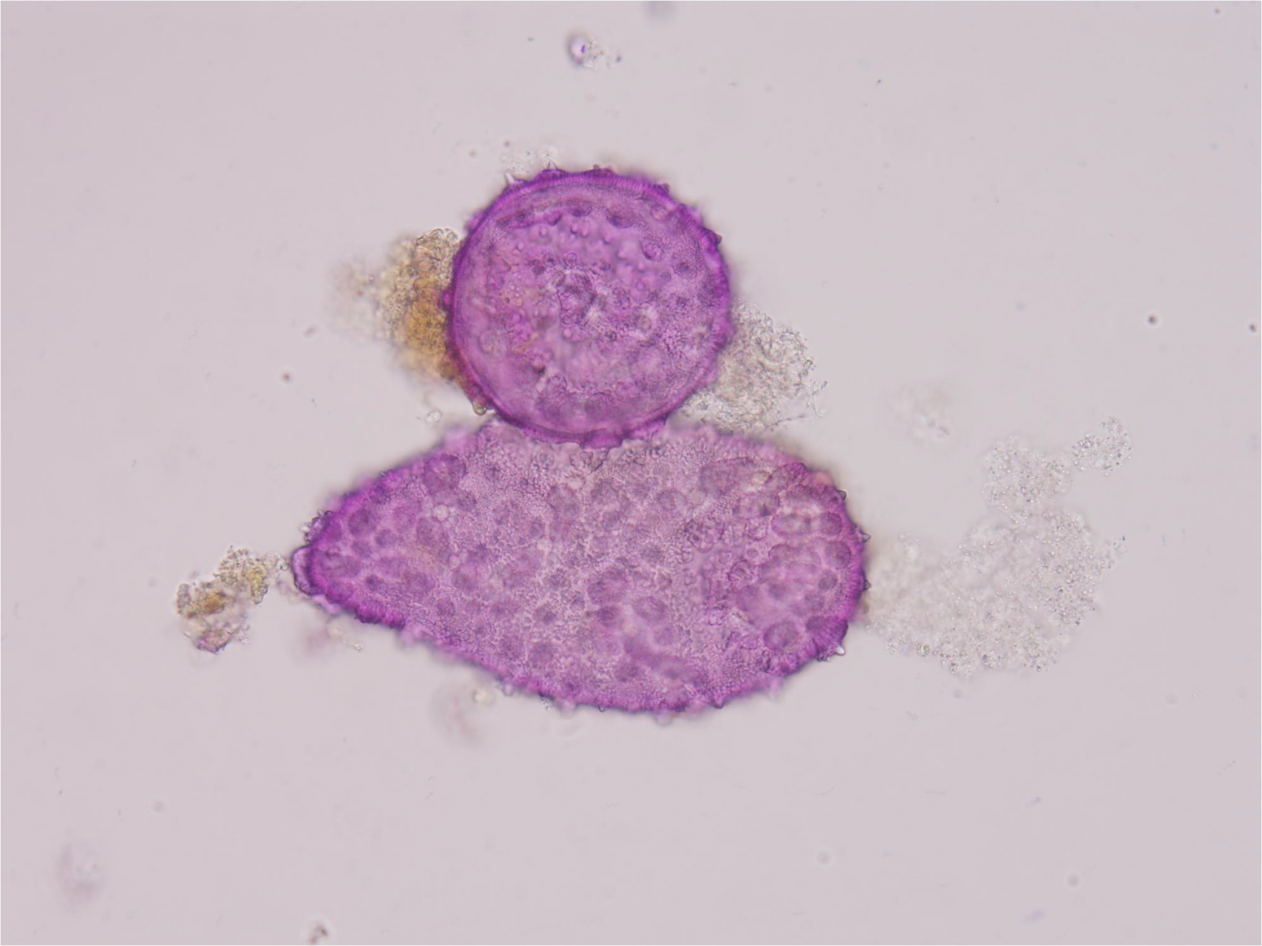
Abnormal meiosis behavior of the hybrid with several micronuclei of various sizes (different polyads) in telophase II.

**Fig 5:**
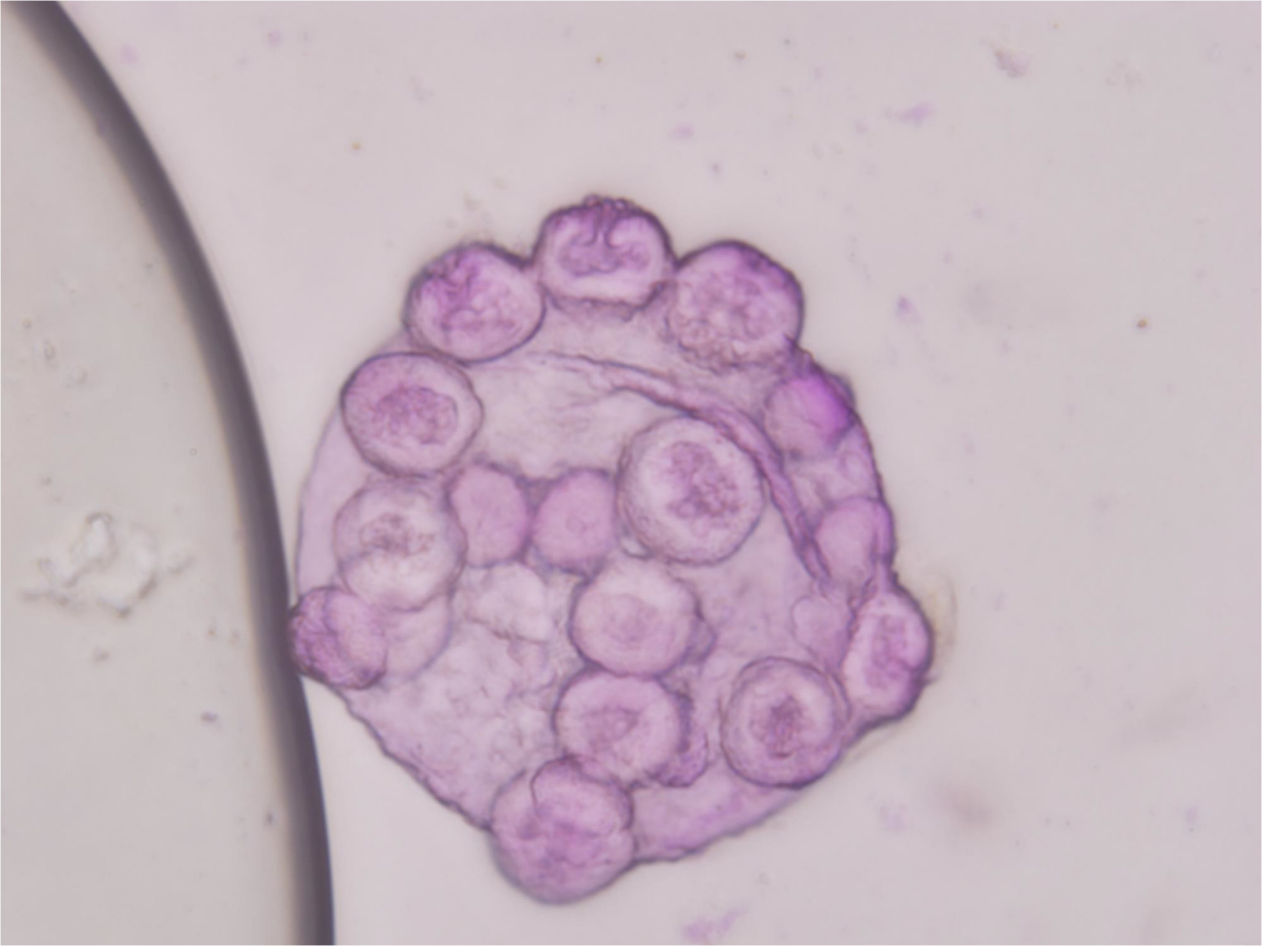
Various abnormal pollen grains of this triple hybrid.

In this study, the meiotic behavior in three parents was quite normal in the first division and completely normal in the second division, generating about 100% of normal tetrads. Observations were also made on chromosome morphology and behavior during meiosis for this triple hybrid. After the occurrence of an irregular first cytokinesis, the meiocytes progressed normally to the second division, generating various numbers of unbalanced microspores mainly because anaphase chromosome movements were blocked. As a consequence of this abnormality in chromosome orientation, a few or several micronuclei of various sizes were observed inside the cells in telophase I and II with diads, triads and different polyads (Fig 4). Finally, various abnormal pollen grains were appeared like cracked pollen, big oval-shaped pollen, under-developed pollen and so on (Fig 5). Only a small portion of pollen grains are normal style. These meiotic observations are similar to those previously reported by Rieseberg [10]. It was further proved that this hybrid is actually wide cross product from different three parents and maybe it has a long way to get into harmonious genome interaction from B, A, D and C. But it gives us suggestive thinking that it is possible to produce a novel germplasm recombining three parents characters through the production of trispecific hybrid.

### SSR molecular analysis

In this paper, the hybrid and its three parents were analyzed using SSR markers from DNA molecular level. A total of 112 selected polymorphism SSR primers were amplified and 98 pairs were screened effective bands in these four accessions. A total of 230 DNA bands were scored from 98 primers for this hybrid, with loci number ranging from one to six per primer pair. By statistic analysis of amplified bands, the genetic proportion coming from three parents in this hybrid is in Table 2. Among the SSR markers in this trispecific hybrid, 47.8% of them were coming from *G*. *hirsutum*, 14.3% from *G*. *australe*, 7.0% from *G*. *capitis-viridis*. Other 30.9% markers were the new and peculiar ones for this hybrid. Different new polymorphics of molecular markers from its parents resulting from difference of sequences might be due to chromosomal rearrangements in this newly formed hybrid [11]. This trispecific hybrid had the characteristic bands of three parents and accordingly had its parent genetic materials from SSR molecule marker analysis. It was also testified that wild genetic material has been transferred into cultivated cotton and this new germplasm can be incorporated into next cotton breeding program. It was also tested that SSR analyses can provide insight into the genome difference in hybrid identification.

**Table 2:** The resources of the SSR polymorphism in trispecific hybrid.

Materials	Triple hybrid	*G*. *hirsutum*	*G*. *australe*	*G*. *capitis-viridis*	Recombination
SSR bands	230	110	33	16	71
Genetic ratio	—	47.8%	14.3%	7.0%	30.9%

Our examinations from morphology and cytogenetic analysis in meiosis following with SSR identification showed that this hybrid was actually wide cross product from three parents *G*. *hirsutum*, *G*. *australe* and *G*. *capitis-viridis*.

## Discussion

### Speciation following with hybridization and Polyploidy

Polyploidy is a prominent process in plant evolution. Approximately 70% of angiosperms are thought to have experienced one or more episodes of polyploidy at some point in the past [12]. Among the best studied polyploids are many of the world’s leading crops, including cotton, wheat, oat, soybean, peanut, canola, tobacco, coffee, and banana, each of which evolved by the joining of divergent genomes in a common nucleus [13]. The evolution of the genus *Gossypium* (cotton) has included a very successful experiment in polyploid formation by hybridization between a maternal Old World ‘‘A” genome taxon resembling *G*. *herbaceum* (2*n* = 2*x* = 26) and paternal New World ‘‘D” genome taxon resembling *G*. *raimondii* [14] or *G*. *gossypioides* [15] (both 2*n* = 2*x* = 26) [16]. Similarly, the reunion of genomes through hybridization and allopolyploidy is conservatively estimated to account for 2–4% of speciation events in flowering plants and 7% in ferns [17], and most plants appear to be paleopolyploids [18]. Hybridization and introgression may play an important role in evolution and often serves as a repair or replacement strategy, rather than solely as a mechanism for the development and/or acquisition of novel traits [19]. Cells differing only by their ploidy are identical in terms of DNA sequence information and relative gene dosage, and yet are often quite different in terms of physiology, morphology, and behavior [20].

### Genetic basis of morphological characteristic

The justification for the use of these characters is the assumption that they have a complex genetic basis of this triple hybrid. Rieseberg [21] indicates that the genetic basis of morphological differences between wild plant species possibly controlled by a single major locus plus modifiers. Vlot et al [22] found a molecular marker locus associated with the major gene controlling pappus part number. Doebley et al [23] also investigated that the genetic control of morphological traits differentiating maize from its wild ancestor fit the general model of a single major locus with large effects plus modifiers. These contrasts indicate polyploidy speciation in plants is accompanied by a diverse array of molecular evolutionary phenomena, which will vary among both genomic constituents and taxa [24], also between some wide cross hybrids and their parents. A better understanding of the number of loci responsible for species differences would greatly assist in assessing the reliability of particular morphological characters, reproductive morphology, frequently employed for phylogenetic and taxonomic inference [21] and hybrids identification.

### Genome interaction of distant hybrid

In most cases, divergent genomes do not coexist peacefully upon first contact and hybrids, if formed at all, are inviable or sterile [25]. Although F_1_ sterility or inviability is a common feature of wide interspecific crosses, small introgressions often have indiscernible heterozygous effects while being lethal or sterilizing in the homozygous state [26]. Reproductive barrier formation between newly derived hybrid taxa and their parental species represents a major evolutionary hurdle [10]. Rieseberg [27] studies of the genes causing hybrid incompatibilities is that postzygotic isolation often as a by-product of adaptation. However, natural selection for viability and fertility provides an effective filter that eliminates gametes and individuals carrying maladaptive changes and favors those inheriting advantageous changes [18]. The genomic changes appeared to be largely complete by the third generation following polyploidy formation, and were accompanied by an increase in seed fertility and a reduction of irregular chromosome pairing in meiosis [28].

In this study, we hope to incorporate *G*. *australe* and *G*. *capitis-viridis* genetic material into upland cotton with some novel traits from the species, such as insect and disease resistance from *G*. *capitis-viridis* and low gossypol seed & high-gossypol plant trait introgressed from *G*. *australe*. Its other excellent agronomic traits need our further identification.
